# Potential Mediators between Fibromyalgia and C-Reactive protein: Results from a Large U.S. Community Survey

**DOI:** 10.1186/s12891-017-1641-y

**Published:** 2017-07-07

**Authors:** Termeh Feinberg, Usha Sambamoorthi, Christa Lilly, Kim Karen Innes

**Affiliations:** 1 Department of Family and Community Medicine, Center for Integrative Medicine, University of Maryland School of Medicine, 520 W. Lombard St., East Hall, Baltimore, MD 21201-1603 USA; 2 Department of Epidemiology, West Virginia University School of Public Health, P.O. Box 9190, Morgantown, WV 26506-9190 USA; 3 Department of Pharmaceutical Systems and Policy, West Virginia University School of Pharmacy, P.O. Box 9500, Morgantown, WV 26506-9500 USA; 4 Department of Biostatistics, West Virginia University School of Public Health, P.O. Box 9190, Morgantown, WV 26506-9190 USA; 5 Center for the Study of Complementary and Alternative Therapies, University of Virginia Health System, P.O. Box 800782, McLeod Hall, Charlottesville, VA 22908-0782 USA

**Keywords:** Fibromyalgia, C-reactive protein, Inflammation, Body mass index, Comorbidity, Mediator, Epidemiology

## Abstract

**Background:**

Fibromyalgia, a potentially debilitating chronic pain syndrome of unknown etiology, may be characterized by inflammation. In this study, we investigated the relation of FMS to serum C-reactive protein (CRP) in a large population of adults (18+) and investigated the influence of other factors on this relationship, including BMI, comorbidities, as well as mood and sleep disturbance.

**Methods:**

Participants were 52,535 Ohio Valley residents (Fibromyalgia *n* = 1125). All participants completed a comprehensive health survey (2005–2006) part of the C8 Health Project; serum levels of CRP were obtained, as was history of Fibromyalgia physician diagnosis. Logistic and linear regressions were used for this cross-sectional analysis.

**Results:**

Mean CRP was higher among participants reporting Fibromyalgia than those without (5.54 ± 9.8 vs.3.75 ± 7.2 mg/L, *p* < .0001)). CRP level showed a strong, positive association with FMS (unadjusted odds ratio (OR) for highest vs. lowest quartile = 2.5 (CI 2.1,3.0;p for trend < .0001)); adjustment for demographic and lifestyle factors attenuated but did not eliminate this association (AOR for highest vs. lowest quartile = 1.4 (CI 1.1,1.6;p for trend < .0001)). Further addition of body mass index (BMI) and comorbidities to the model markedly weakened this relationship (AORs, respectively, for highest vs lowest CRP quartile = 1.2 (CI 1.0,1.4) and 1.1 (CI 0.9,1.3). In contrast, inclusion of mood and sleep impairment only modestly reduced the adjusted risk estimate (AORs for highest vs. lowest quartile = 1.3 (CI 1.1,1.5) for each)).

**Conclusions:**

Findings from this large cross-sectional study indicate a significant positive cross-sectional association of Fibromyalgia to serum C-reactive protein may be explained, in part, by BMI and comorbidity. Prospective research is needed to confirm this, and clarify the potential mediating influence of obesity and comorbid conditions on this relationship.

## Background

Chronic pain, defined as ongoing or recurrent pain that extends beyond the usual course of acute illness or injury for at least three to six months, is debilitating and costly. Over 20% of adults experience chronic pain at some point in their lives [1]. Fibromyalgia syndrome (FMS) is a rheumatologic chronic pain syndrome affecting approximately 0.5–5% of populations in developed countries [2], including 1.75% of those in the U.S. [3]. FMS is characterized by a constellation of somatic symptoms that are typically present in addition to widespread pain (e.g., fatigue, sleep disturbance, memory, and mood problems), for which no clear cause can be found [4]. FMS is typically accompanied by morning stiffness, and sensitivity to loud noises, bright lights, and temperature extremes; women with FMS often report painful menstrual periods [5]. Although FMS affects both sexes and people of all ages, the majority (80–90%) have been Caucasian [5, 6] middle-aged women [5]. Family members of FMS patients are at a higher risk for FMS [7]; the cause is unknown.

While the etiology of FMS remains poorly understood, the widespread pain of FMS is thought to reflect abnormal central nervous system sensory information processing, with altered function in pain pathways and neuroendocrine disturbance [4]; Inflammatory processes may also play a significant role in the pathogenesis of FMS [8]. Although several MicroRNAs have been associated with FMS and symptom severity [9], diagnosis of FMS remains challenging, and there are currently no definitive diagnostic laboratory tests for the disease [10].

C-reactive protein (CRP), previously considered a biomarker of underlying infection or tissue injury [11], is now also believed to reflect chronic systemic inflammation [12]. CRP is considered a reliable proinflammatory biomarker [11] and is often included as part of the diagnostic laboratory workup for many rheumatological conditions [11]. While CRP is often included in the diagnostic workup for FMS, the relationship of CRP to FMS has not been clearly established.

Inflammation, as a characteristic reaction of tissues to injury or disease marked by physical swelling, redness, heat, and pain upon clinical examination, is not a classical symptom of FMS [10]. Inflammatory cytokines promote the development of contralateral hyperalgesia (an extreme, exaggerated reaction to pain) and allodynia (central pain sensitization following painful, often repetitive stimulation) [13]. FMS is typically characterized by both [14], and most [15–28], but not all recent studies [23, 29] have suggested a possible link between systemic inflammation and FMS. Of these, five studies measured CRP [24, 26–28, 30]; four suggested a positive association between CRP and FMS [24, 26–28]. However, to date, only one large, cross-sectional, population-based study has examined the association of CRP to FMS [27]. The study excluded women, and combined FMS with other pain syndromes, rendering assessment of the specific relationship between CRP and FMS difficult.

Moreover, to our knowledge, no studies to date examining the association of CRP to FMS have investigated the potential mediating effects of sleep or mood disturbance, factors linked to both elevated CRP levels and FMS [31–34]. Few have assessed the influence of elevated BMI [33, 35], comorbidities, and other correlates [24]. This large, population-based study will fill the gaps in our understanding of the potential influence(s) of mood, sleep, BMI, and comorbid conditions on the relationship between CRP and FMS.

## Methods

In this study, we investigated the relation of serum CRP levels to FMS in a large population of US adults.

### Data source

This cross-sectional study used data from the C8 Health Project, which was conducted as part of the settlement of a class-action lawsuit stemming from drinking water contamination by Perfluorooctanoic Acid (PFOA) released from the DuPont Washington Works Plant near Parkersburg, WV, USA [36]. Data collection was conducted in 2005–2006 on individuals living or working in 6 PFOA-contaminated public water districts in West Virginia (WV) and Ohio (including those exposed to contaminated private-well drinking water) since 1951; a total of 69,030 individuals participated in the study, including 81% of eligible adults [36]. Project data collection was administered by an independent company, Brookmar, Inc. (Parkersburg, WV), and was conducted under the authority and supervision of the Wood County, WV, Circuit Court. Participants completed a comprehensive health questionnaire and volunteered a blood sample after completing individual consent forms for both. Demographic data and health survey completion were verified by trained project staff [36]. Project procedures, blood processing and assay methods, along with quality-assurance measures, have been described in detail elsewhere [36]. Briefly, following collection of each blood sample, serum was separated from red blood cells into single-use aliquots by centrifusion, and was refrigerated at individual data collection sites until daily pickup from a large, independent, accredited clinical diagnostic laboratory (LabCorp, Inc., Burlington, NC, USA). Samples were transported to a regional processing center (LabCorp, Inc., Columbus, OH) where they underwent analysis by latex immunoturbidimetry on a COBAS Integra 800 (Roche, Germany). The West Virginia University (WVU) IRB permitted access to the de-identified data by WVU investigators. Demographic, lifestyle, and health characteristics were determined via self-report; diagnoses of certain disorders, including diabetes and cardiovascular diseases, were further verified via chart review.

### Study population

**Fig. 1:**
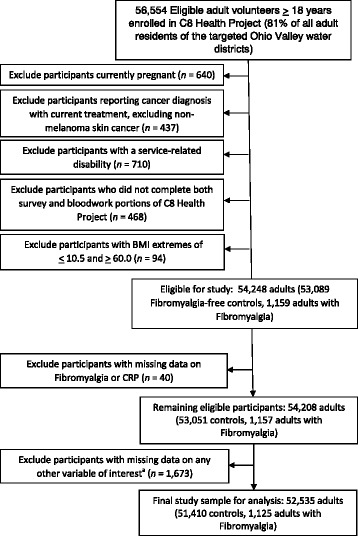
^a^: with exceptions of income and current menstruation, which contained ≥10% of missing values; these were considered separate categories for analysis

Our analysis excluded participants who were missing data on age or <18 years of age (*n* = 12,471, 18.1%), pregnant (*n* = 640, 1.1%); those reporting a cancer diagnosis and receiving treatment for diagnosed cancer other than non-melanoma skin cancer (*n* = 437, 0.77%) to eliminate potential bias introduced by varied CRP levels as a result of chemotherapy treatment; those with service-related disabilities (*n* = 710, 1.3%); and those who did not complete both the survey and blood work (*n* = 468, 0.83%). Participants with extreme body mass index (BMI) values (<10.5 and >60.0) were also excluded to eliminate potential information bias (*n* = 94, 0.17%). Further exclusion of persons with missing data on CRP and FMS (*n* = 40, 0.002%) and other covariates of interest (*n* = 1673, 3.1%), with the exception of covariates for which missing data on >10% of participants occurred (for which a ‘missing’ category was included in analysis), yielded a final study sample of 52,535, including 51,410 FMS-free controls and 1125 adults with FMS (Fig. 1).

### Outcome variable

Our primary, dichotomous outcome variable was FMS diagnosis, which was ascertained via self-report response to the question “Have you ever been diagnosed with Fibromyalgia?”

### Exposure variable

Our primary exposure variable was serum level of inflammatory marker CRP (mg/L). The normal CRP range in the general population is considered 0.0–5.0 mg/L [28]. Further, in healthy young adult volunteer blood donors, the median concentration of CRP is 0.8 mg/L, the 90th centile is 3.0 mg/L, and the 99th centile is 10 mg/L; following an acute-phase stimulus, values may increase from less than 50 μg/L to more than 500 mg/L [11]. C8 Health Project coordinators recoded CRP values below the level of detection to 50% of the lowest level (i.e., “<0.3” → 0.15). Likewise, values above the level of detection were recoded to 50% above the maximum level.

### Covariates

In addition to age, gender, race, education, employment, marital status, income, alcohol or tobacco use, and exercise program status, comorbid conditions were also selected a priori as covariates if known or suspected to be associated with FMS and/or CRP. These included BMI [37], autoimmune conditions [38], osteoarthritis [39], kidney [40], respiratory [41], cardiovascular [40], liver [42], and endocrine [40] disease, diabetes [43], severe allergies [40] and sinus disease [44], stomach conditions [40, 42], and headache [45]. PFOA serum level and reproductive factors [46] were also examined as covariates.

### Potential mediating and modifying factors

Potential mediating and modifying factors included fatigue, sleep impairment [42], mood disturbance [32, 40, 42], age [5], gender [5], obesity [35], tobacco use [47], statin use [48], and reproductive factors [46].

### Statistical analysis

We conducted complete-case analyses using SAS 9.4 (Cary, NC, USA). Logistic regression analyses were used to evaluate the independent association of CRP level to FMS status, and to assess the influence of potential confounding, mediating, and modifying factors. The primary explanatory variable of interest, CRP, was analyzed as a continuous and categorical variable (study population quartiles, with the lowest percentile group used as the referent). Linear trends for CRP quartiles were assessed using polynomial contrasts. Differences between participants missing any data were assessed using logistic regression. Cox-Snell R^2^ values measured the predictive power of models. All *p*-values shown are two-sided.

All demographic characteristics, in addition to lifestyle factors significantly differing by FMS, were controlled for in multivariate models. BMI was categorized using the National Institutes of Health clinical classifications (scores of <25 = ‘Underweight or Normal weight’; 25–29.9 = ‘Overweight’; 30–34.9 = ‘Obese Class 1’; and, 35 + =‘Obese Classes 2/3’) [49].

We evaluated the influence of both specific comorbid conditions and total number of comorbid conditions on the relation of CRP to FMS. A comorbidity index was created based on number of chronic comorbid conditions reported; these included: autoimmune disorders (defined as having any diagnoses of immune disease, lupus, or rheumatoid arthritis), osteoarthritis, allergies or frequent sinusitis, kidney disease, respiratory conditions (asthma, emphysema, chronic obstructive pulmonary disorder, bronchitis), heart disease, liver disease, endocrine disorders (thyroid, Addison’s, or Cushing’s disease), diabetes, or frequent headaches. This index was evaluated as both a continuous and categorical variable (‘none’, ‘1 comorbidity’, 2 comorbidities’, and ‘3+ comorbidities’).

We assessed potential mediating influences of sleep impairment and mood disturbance. A composite sleep quality variable, with higher scores indicating poorer sleep quality, was derived from responses to individual questions regarding the frequency of short sleep, fitful sleep, insomnia, and/or daytime somnolence (scored as follows: 3 = ‘frequently’, 2 = ‘sometimes’, 1 = ‘rarely’, and 0 = ‘never’, for each). Mood disturbance was also assessed as a composite variable from responses to four individual questions, with higher scores indicating frequent mood swings, irritability, fatigue, and/or inability to concentrate (where 3 = ‘frequently’, 2 = ‘sometimes’, 1 = ‘rarely’, and 0 = ‘never’).

Additionally, we assessed the potential modifying influence of age (<45 vs. ≥45 years), tobacco use (‘current’/‘not currently using tobacco’), gender, obesity (BMI < 30 vs. ≥30) current menstruation (yes/no), menopausal status (peri- or post-menopausal/premenopausal), statin use (yes/no), and sleep impairment and mood disturbance (scores of <6 vs. ≥ 6, respectively) on the association between CRP and FMS.

We also conducted separate ancillary analyses adjusting for statin use (yes/no), PFOA serum level (ng/mL, evaluated as quartiles), and reproductive characteristics (women only) to determine the potential confounding influence of these factors on any observed association between CRP and FMS. Reproductive factors assessed included menopausal status (‘pre-menopausal’; ‘peri-or post-menopausal’; or ‘unsure’), parity (number of pregnancies), and age at menarche (‘16+ years of age’/‘other age or unsure’).

The first regression model assessed the crude association of CRP levels to reported FMS diagnosis. The second model was adjusted for demographic and lifestyle covariates, while additional models adjusted for BMI and comorbid conditions, as well as sleep and mood disturbance (separately and combined, respectively). We conducted additional analyses to evaluate the potential confounding influence of statin use, PFOA serum level, and (in female participants) reproductive history (i.e., menopausal status, age at menarche, and parity).

The potential mediating effects of BMI and comorbid conditions, as well as sleep impairment and mood disturbance were examined using separate logistic regression models. We also conducted ancillary analyses excluding autoimmune disorders rheumatoid arthritis, lupus, and self-reported ‘previous diagnosis of immune disorder.’ To evaluate the potential modifying effects of age, smoking, gender, obesity, current menstruation, menopausal status, statin use, sleep impairment, and mood disturbance, we conducted multivariable analyses; interactions were assessed by including the corresponding multiplicative-interaction term in the statistical models.

## Results

Relative to participants with complete data, those with missing data on key covariates had a higher number of comorbidities and were more likely to be poorly educated or to be retired, disabled, or unemployed (*p’s* ≤ .002), but did not differ in other factors. Demographic and lifestyle characteristics are displayed in Table 1. Study participants were predominantly white (97.2%), female (52.4%), and married or cohabitating (68.9%). Participant age ranged from 18.0 to 105.2 years, averaging 45.3 (SD = 16.1) years. Most participants were employed or students (64.1%) and overweight (69.0% BMI ≥ 25, mean BMI = 28.5, SD = 6.3), while nearly half did not currently consume alcohol (51.1%) and never smoked (43.5%). CRP serum levels varied from 0.15 to 250.6 (mean CRP level = 3.79, SD = 7.2) mg/L. Additionally, PFOA was not significantly associated with CRP after adjustment with all factors used in other models (*p* = .084).

**Table 1:** Characteristics of a US adult population by Fibromyalgia (FMS) status, Adjusted for Demographic and Lifestyle factors, C8 Health Project, 2005–2006

Characteristic	Total Population	FMS	Non-FMS	Adjusted Odds Ratio^a^
	N (%) / Mean (SD)			OR (CI), *p*-value
Total	52,535	1125 (2.1%)	51,410 (97.9%)	−
Age (years)	45.25 (16.14)	51.14 (11.58)	45.12 (16.20)	1.02 (1.02–1.03), <.0001^*^
Age				
18–24 (ref)	6540 (12.5%)	16 (1.4%)	6524 (12.7%)	−
25–44	20,178 (38.4%)	300 (26.7%)	19,879 (38.7%)	3.89 (2.30–6.56), <.0001^*^
45–64	19,098 (36.4%)	689 (61.2%)	18,409 (35.8%)	8.28 (4.91–13.95), <.0001^*^
65+	6719 (12.8%)	120 (10.7%)	6599 (12.8%)	4.37 (2.50–7.64), <.0001^*^
Gender				
Male (ref)	25,002 (47.6%)	90 (8.0%)	24,912 (48.5%)	−
Female	27,533 (52.4%)	1035 (92.0%)	26,498 (51.5%)	10.68 (8.53–13.37), <.0001^*^
Race/Ethnicity				
Non-Hispanic White	51,092 (97.2%)	1098 (97.6%)	49,994 (97.2%)	1.09 (0.73–1.62), .683
Other Race (ref)	1443 (2.8%)	27 (2.4%)	1416 (2.8%)	−
Education				
< 12th grade (ref)	6261 (11.9%)	106 (9.4%)	6155 (12.0%)	−
HS/GED	22,353 (42.6%)	445 (39.6%)	21,908 (42.6%)	1.57 (1.25–1.96), .0001^*^
Some College	17,081 (32.5%)	442 (39.3%)	16,639 (32.4%)	2.22 (1.75–2.82), <.0001^*^
Bachelor’s degree+	6840 (13.0%)	132 (11.7%)	6708 (13.1%)	1.91 (1.42–2.56), <.0001^*^
Employment				
Employed/Student (ref)	33,682 (64.1%)	482 (42.8%)	33,200 (64.6%)	−
Retired/Unemployed	9057 (17.2%)	144 (12.8%)	8913 (17.3%)	1.44 (1.16–1.79), .0008^*^
Disabled	3357 (6.4%)	252 (22.4)	3105 (6.0%)	5.70 (4.73–6.87), <.0001^*^
Homemaker	5979 (11.4%)	233 (20.7%)	5746 (11.2%)	1.50 (1.26–1.78), <.0001^*^
Other	460 (0.88%)	14 (1.2%)	446 (0.9%)	2.23 (1.28–3.88), .004^*^
Marital Status				
Married/Cohabitating (ref)	36,198 (68.9%)	836 (74.3%)	35,362 (68.8%)	−
Single	8729 (16.6%)	60 (5.3%)	8669 (16.9%)	0.63 (0.47–0.84), .002^*^
Divorced/Sep/Widow	7608 (14.5%)	229 (20.4%)	7379 (14.4%)	0.85 (0.71–1.01), .058
Household Income				
Don’t know/Missing	5335 (10.2%)	92 (8.2%)	5243 (10.2%)	0.85 (0.66–1.10), .217
< $20,000 (ref)	12,308 (23.4%)	288 (25.6%)	12,020 (23.4%)	−
$20,000–40,000	14,326 (27.3%)	301 (26.8%)	14,025 (27.3%)	1.03 (0.86–1.24), .759
$40,001–70,000	13,559 (25.8%)	291 (25.9%)	13,268 (25.8%)	1.05 (0.86–1.29), .619
$70,000+	7007 (13.3%)	153 (13.6%)	6854 (13.3%)	1.09 (0.85–1.39), .514
Alcohol				
Don’t drink (ref)	26,847 (51.1%)	722 (64.2%)	26,125 (50.8%)	−
Currently Drink	25,688 (48.9%)	403 (35.8%)	25,285 (49.2%)	0.86 (0.75–0.98), .023^*^
Tobacco User				
Never (ref)	22,859(43.5%)	567 (50.4%)	22,292 (43.4%)	−
Current	16,691 (31.8%)	274 (24.4%)	16,417 (31.9%)	0.91 (0.78–1.07), .243
Former	12,985 (24.7%)	284 (25.2%)	12,701 (24.7%)	1.06 (0.91–1.23), .441
Exercise Program				
No regularity (ref)	36,113 (68.7%)	762 (67.7%)	35,351 (68.8%)	−
Regular exercise	16,422 (31.3%)	363 (32.3%)	16,059 (31.2%)	−
Menopause occurred	10,526 (38.2%)	631 (61.0%)	9895 (37.3%)	1.82 (1.49, 2.21), <.0001^*^

Note: Column Percentages shown

*Significant at *p* < 0.05

^a^Model adjusted for demographic covariates Age, Gender, Marital Status, Employment, Education level, White Race, and Household Income; Also adjusted for lifestyle covariates Tobacco Use and Current Alcohol consumption

FMS was present in 2.1% of the study population (*n* = 1125). After adjustment for demographic and lifestyle factors, the odds of FMS increased 2% for every year-unit increase in age (Adjusted odds ratio (AOR) = 1.02, 95% confidence interval (CI): 1.02, 1.03 (Table 1)), with a significantly higher mean age among FMS cases (*M* = 51.1, SD = 11.6) compared to controls (*M* = 45.1, SD = 16.2). Those aged 45–64 years were 8.3 times more likely (AOR = 8.28, CI 4.91, 13.95) than those in the age group 18–24 years to have FMS. Those with FMS were nearly 11 times more likely to be female (AOR = 10.68, CI 8.53, 13.37). Participants who were employed or students were less likely to have FMS than all others, as were those with <12th grade education, compared to those with higher educational attainment. Relative to participants who were married or cohabitating, those who were single were almost 40% less likely to report a diagnosis of FMS (AOR = 0.63, CI 0.47, 0.84). Participants who reported current consumption of alcohol were slightly less likely to report a diagnosis of FMS (35.8% vs. 49.2%, respectively; AOR = 0.86, CI 0.75, 0.98), and women who had experienced menopause were about 80% more likely to have an FMS diagnosis compared to pre-menopausal women (AOR = 1.82, CI 1.49, 2.21).

Health characteristics of the study population are detailed in Table 2. Mean BMI was significantly higher among FMS cases (*M* = 30.47, SD = 7.2) than controls (*M* = 28.49, SD = 6.3), and was significantly and positively associated with FMS after adjustment for demographic and lifestyle factors (AOR per unit BMI increase = 1.02, CI 1.01, 1.03). Relative to those with a BMI <25, participants who were obese were significantly more likely to have FMS (AOR = 1.24, CI 1.02, 1.49; and, AOR = 1.43, CI 1.18, 1.74, for BMI 30–35 and BMI >35, respectively). There was a 60% increase in FMS diagnosis for each additional comorbidity (AOR = 1.60, CI 1.54, 1.67 (Table 2); participants reporting a diagnosis of 3 or more comorbidities were 10.5 times more likely to have FMS (AOR = 10.46, CI 7.90, 13.84) compared to those with no comorbidities. FMS was likewise strongly and positively associated with most chronic conditions evaluated, including in order of decreasing magnitude osteoarthritis (AOR = 3.86, CI 3.34, 4.45), autoimmune disease (AOR = 3.43, CI 2.89, 4.06), allergies/chronic sinusitis (AOR = 2.91, CI 2.51, 3.38), frequent headaches (AOR = 2.03, CI 1.78, 2.31), and kidney (AOR = 1.72, CI 1.47, 2.01), respiratory (AOR = 1.89, CI 1.64, 2.17), endocrine (AOR = 1.82, CI 1.57, 2.12), and liver (AOR = 1.51, CI 1.06, 2.16) diseases.

**Table 2:** Health Characteristics of a US adult population by Fibromyalgia (FMS) status, Adjusted for Demographic and Lifestyle factors, C8 Health Project, 2005–2006

Characteristic	Total Population	FMS	Non-FMS	Adjusted Odds Ratio^a^
	N (%) / Mean (SD)			OR (CI), *p*-value
BMI+	28.53 (6.31)	30.47 (7.22)	28.49 (6.28)	1.02 (1.01–1.03), .001^*^
≤ 24.99 (ref)	16,258 (31.0%)	279 (24.8%)	15,979 (31.1%)	−
25.5–29.99	18,200 (34.6%)	320 (28.4%)	17,880 (34.8%)	1.11 (.932–1.31), .248
30–34.9	10,704 (20.4%)	251 (22.3%)	10,453 (20.3%)	1.24 (1.02–1.49), .028^*^
35+	7373 (14.0%)	275 (24.4%)	7098 (13.8%)	1.43 (1.18–1.74), .0003^*^
Number of Comorbidities	1.29 (1.27)	2.78 (1.59)	1.25 (1.24)	1.60 (1.54–1.67), <.0001^*^
Comorbidity Index				
None (ref)	16,778 (31.9%)	58 (5.2%)	16,720 (32.5%)	−
1	16,999 (32.4%)	177 (15.7%)	16,822 (32.7%)	2.38 (1.77–3.21), <.0001^*^
2	10,535 (20.1%)	301 (26.8%)	10,234 (19.9%)	5.16 (3.88–6.86), <.0001^*^
3+	8223 (15.7%)	589 (52.4%)	7634 (14.9%)	10.46 (7.90–13.84), <.0001^*^
*Autoimmune disorder*	2192 (4.2%)	220 (19.6%)	1972 (3.8%)	3.43 (2.89–4.06), <.0001^*^
*Osteoarthritis*	4093 (7.7%)	411 (35.5%)	3782 (7.1%)	3.86 (3.34–4.45), <.0001^*^
*Allergies or Chronic Sinusitis*	24,470 (46.6%)	893 (79.4%)	23,577 (45.9%)	2.91 (2.51–3.38), <.0001^*^
*Kidney disorder*	5202 (9.9%)	226 (20.1%)	4976 (9.7%)	1.72 (1.47–2.01), <.0001^*^
*Respiratory disease*	7385 (14.1%)	342 (30.4%)	7043 (13.7%)	1.89 (1.64–2.17), <.0001^*^
*Cardiovascular disease*	4661 (8.9%)	148 (13.2%)	4513 (8.9%)	1.25 (1.03–1.51), .027^*^
*Liver disease*	684 (1.3%)	38 (3.4%)	646 (1.3%)	1.51 (1.06–2.16), .022^*^
*Endocrine Disorder*	4083 (7.8%)	264 (23.5%)	3819 (7.4%)	1.82 (1.57–2.12), <.0001^*^
*Diabetes*	4778 (9.1%)	157 (14.0%)	4621 (9.0%)	.978 (.813–1.18), .810
*Frequent Headache*	10,105 (19.2%)	439 (39.0%)	9666 (18.8%)	2.03 (1.78–2.31), <.0001^*^
Sleep Impairment Score^b^	4.00 (3.56)	7.32 (3.29)	3.93 (3.53)	1.23 (1.21–1.25), <.0001^*^
Mood Disturbance Score^m^	4.59 (3.70)	7.25 (3.40)	4.53 (3.68)	1.19 (1.17–1.21), <.0001^*^

Note: Column Percentages shown

*Significant at *p* < 0.05

^a^Model adjusted for demographic covariates Age, Gender, Marital Status, Employment, Education level, White Race, and Household Income; Also adjusted for lifestyle covariates Tobacco Use and Current Alcohol consumption

^b^Sleep impairment derived from responses to four individual questions regarding the frequency of short sleep, fitful sleep, insomnia and/or daytime somnolence; higher score indicates increased frequency of impairment

^m^Mood disturbance derived from responses to four individual questions regarding frequent mood swings, irritability, fatigue and/or inability to concentrate; higher score indicates increased frequency of disturbance

FMS cases reported higher sleep (*M* = 7.3, SD = 3.3 vs. *M* = 3.9, SD = 3.5) and mood disturbance scores (*M* = 7.3, SD = 3.4 vs. *M* = 4.5, SD = 3.7) than did controls (Table 2). After adjustment for demographic and lifestyle factors, FMS remained strongly and positively related to scores for both sleep (AOR = 1.23, CI 1.21, 1.25), and mood disturbance (AOR = 1.19, CI 1.17, 1.21).

### Relation of CRP to FMS

Mean CRP (mg/L) was significantly higher among FMS cases (*M* = 5.54, SD = 9.8) compared to controls (*M* = 3.75, SD = 7.2) (Table 3). CRP serum level showed a positive association with FMS (unadjusted OR for highest vs. lowest quartile = 2.50, CI 2.10, 2.97; P for trend <.0001); adjustment for demographic and lifestyle factors substantially attenuated but did not eliminate this association (AOR for highest vs. lowest quartile = 1.35, CI 1.13, 1.62; P for trend <.0001). Analysis of CRP as a continuous variable yielded similar findings, with odds of FMS increasing by 2% for each one mg/L of CRP rise (unadjusted OR = 1.02, CI 1.01, 1.02); adjustment for demographic and lifestyle factors slightly attenuated this association (AOR = 1.01, CI 1.00, 1.01).

**Table 3:** Model Statistics assessing the association between Fibromyalgia (FMS) and blood serum C-reactive Protein (CRP) quartile adjusting for BMI and Comorbidity index in a US adult population, 2005–2006 (*N* = 52,535)

Characteristic	Total	FMS	Non-FMS	Models				
				Odds Ratio (CI) (*p*-value)				
	N (%) / Mean (SD)			Crude^e^	Adjusted for Demographic and Lifestyle Factors (Model 1)^f^	Model 1 ± BMI^g^	Model 1 ± comorbidities^h^	Model 1 ± BMI & comorbidities^r^
CRP level (mg/L)	3.79 (7.22)	5.54 (9.77)	3.75 (7.15)	1.02 (1.01–1.02) (<.0001^*^)	1.01 (1.00–1.01) (.004^*^)	1.01 (1.00–1.01) (.059)	1.00 (.997–1.01) (.267)	1.00 (.997–1.01) (.279)
CRP Quartile 1^a^ (ref)	14,516 (27.6%)	192 (17.1%)	14,324 (27.9%)	—	—	—	—	—
Quartile 2^b^	12,221 (23.3%)	214 (19.0%)	12,007 (23.4%)	1.33 (1.09–1.62) (.004^*^)	1.13 (0.92–1.38) (.243)	1.07 (0.88–1.32) (.473)	1.04 (0.85–1.28) (.686)	1.03 (0.84–1.27) (.750)
Quartile 3^c^	12,755 (24.3%)	297 (26.4%)	12,458 (24.2%)	1.78 (1.48–2.14) (<.0001^*^)	1.23 (1.02–1.48) (.032^*^)	1.12 (0.92–1.37) (.244)	1.08 (0.89–1.30) (.452)	1.06 (0.87–1.29) (.576)
Quartile 4^d^	13,043 (24.8%)	422 (37.5%)	12,621 (24.6%)	2.50 (2.10–2.97) (<.0001^*^)	1.35 (1.13–1.62) (.0009^*^)	1.17 (0.96–1.42) (.120)	1.10 (0.92–1.32) (.310)	1.07 (0.88–1.30) (.510)
Wald X^2^, *p* for trend^t^				118.42, (<.0001*)	17.48, (<.0001*)	4.02, (.045*)	3.44, (.064)	1.23, (.269)

*Significant at *p* < 0.05

^a^C-reactive protein.15–.80 mg/L

^b^C-reactive protein.81–1.80 mg/L

^c^C-reactive protein 1.81–4.20 mg/L

^d^C-reactive protein 4.21–250.6 mg/L

^t^Linear trend

^e^ (R^2=^=.003)

^f^Full Model adjusted for demographic covariates Age, Gender, Marital Status, Employment, Education level, White Race, and Household Income; Also adjusted for lifestyle covariates Tobacco Use and Current Alcohol consumption (R^2^ = .031)

^g^(R^2^ = .031)

^h^Model adjusted for Comorbidity index; index included diabetes, other endocrine disorders (thyroid, Addison’s, and Cushing’s disease), kidney disease, respiratory conditions (asthma, emphysema, chronic obstructive pulmonary disorder, bronchitis), osteoarthritis, heart disease, liver disease, autoimmune disorders (defined as having any diagnoses of immune disease, lupus, or rheumatoid arthritis), sleep apnea, irritable bowel syndrome, allergies or frequent sinusitis, or frequent recurrent headache (R^2^ = .041)

^r^(R^2^ = .041)

The addition of BMI and comorbidities to the model further weakened the relationship between CRP and FMS (AORs for highest vs. lowest CRP quartile = 1.17 (CI 0.96, 1.42) and 1.10 (CI 0.92, 1.32), for BMI and comorbidities, respectively; and, combined OR = 1.07 (CI 0.88, 1.30)) suggesting that these factors may at least partially explain the observed associations (Table 3). The inclusion of mood disturbance and sleep impairment, separately and combined, only slightly attenuated the association of FMS to CRP after adjustment for demographic and lifestyle factors (AOR = 1.01, CI 1.00, 1.01) (Table 4). These findings suggest that any mediating effect of these factors was modest.

**Table 4:** Model Statistics assessing the association between Fibromyalgia (FMS) and blood serum C-reactive Protein (CRP) quartile adjusting for Sleep impairment and Mood disturbance in a US adult population, 2005–2006 (*N* = 52,535)

	Models				
	Odds Ratio (CI) (*p*-value)				
	Crude^e^	Adjusted for Demographic and Lifestyle Factors (Model 1)^f^	Model 1 ± Mood^g^	Model 1 ± Sleep^h^	Model 1 + Mood & Sleep^r^
CRP level (mg/L)	1.02 (1.01–1.02) (<.0001^*^)	1.01 (1.00–1.01) (.004^*^)	1.01 (1.00–1.01) (.021^*^)	1.01 (1.00–1.01) (.035^*^)	1.01 (1.00–1.01) (.031^*^)
CRP Quartile 1^a^ (ref)	—	—	—	—	—
Quartile 2^b^	1.33 (1.09–1.62) (.004^*^)	1.13 (0.92–1.38) (.243)	1.12 (0.91–1.36) (.287)	1.10 (0.90–1.35) (.357)	1.10 (0.90–1.34) (.363)
Quartile 3^c^	1.78 (1.48–2.14) (<.0001^*^)	1.23 (1.02–1.48) (.032^*^)	1.20 (1.00–1.45) (.056)	1.18 (0.98–1.43) (.083)	1.18 (0.97–1.42) (.093)
Quartile 4^d^	2.50 (2.10–2.97) (<.0001^*^)	1.35 (1.13–1.62) (.0009^*^)	1.28 (1.07–1.53) (.007^*^)	1.26 (1.05–1.51) (.011^*^)	1.24 (1.03–1.48) (.021^*^)
Wald X^2^, *p* for trend^t^	118.42, (<.0001*)	17.48, (<.0001*)	11.06, (.0009*)	10.40, (.001*)	8.25, (.004*)

*Significant at *p* < 0.05

^a^C-reactive protein .15–.80 mg/L

^b^C-reactive protein .81–1.80 mg/L

^c^C-reactive protein 1.81–4.20 mg/L

^d^ C-reactive protein 4.21–250.6 mg/L

^t^Linear trend

^e^(R^2=^=.003)

^f^Full Model adjusted for demographic covariates Age, Gender, Marital Status, Employment, Education level, White Race, and Household Income; Also adjusted for lifestyle covariates Tobacco Use and Current Alcohol consumption (R^2^ = .031)

^g^Mood disturbance derived from responses to four individual questions regarding frequent mood swings, irritability, fatigue and/or inability to concentrate (R^2^ = .035)

^h^Sleep impairment derived from responses to four individual questions regarding the frequency of short sleep, fitful sleep, insomnia and/or daytime somnolence (R^2^ = .037)

^r^(R^2^ = .038)

Additional adjustment for statin use, PFOA, and female reproductive characteristics (menopausal status, age at menarche, and parity) did not appreciably change the relationship between CRP and FMS. Similarly, exclusion of those with rheumatoid arthritis (*n* = 1821) and all autoimmune conditions (*n* = 2192) did not appreciably affect risk estimates. Likewise, we found no evidence for a modifying effect of age, tobacco use, gender, obesity, menopausal status, or other factors on the relationship between CRP and FMS.

## Discussion

This is the first large, population-based investigation to examine the relationship between CRP and FMS, to assess the potential influence of BMI and comorbid conditions on this relationship, and to evaluate the potential mediating role of mood and sleep impairment. In this cross-sectional study of a large population in the U.S., CRP serum level showed a positive association with FMS, which remained significant after adjustment for multiple demographic and lifestyle factors, including age, gender, education level, employment, marital status, alcohol, and tobacco use. Adjustment for BMI and comorbid conditions substantially attenuated this relationship. These findings are broadly consistent with those from a recent cross-sectional investigation of 5110 Norwegian men; cases with FMS/Chronic Fatigue Syndrome (CFS) showed a strong, positive relationship to high sensitivity-CRP level (FMS/CFS *M* = 4.79 mg/L; OR for ≥10 mg/L vs. <1 mg/L = 2.6, 95% CI: 1.4, 4.6, *p* = .002; P for trend = .006) after adjustment for age, education, smoking, and cholesterol medication [27]. In contrast, as stated in the introduction, findings from smaller case-control studies examining the association between CRP and FMS have been inconsistent [24, 26, 28, 30], perhaps due to varying sample sizes and differing selection criteria among controls; of the four case-control studies published to date, only one reported significantly higher CRP levels in FMS patients compared to healthy controls after adjustment for age, sex, and race [24]. Mean CRP values found among those with FMS (*M* = 5.54 mg/L) nor non-FMS controls (*M* = 3.75 mg/L) in our study are nearly within the range for normal CRP values (0.0–5.0 mg/L) [28] and also within ranges of those found in most previous studies (mean FMS high-sensitivity CRP range = 2.6–10.6 mg/L; mean FMS CRP range = 1.0–4.7 mg/L) [24, 26, 28, 30]. One exception, however, was that reported by Rus et al. (1.0 ± 0.75) in a Spanish population of women with FMS [28]; in addition to differences in CRP by BMI status, other possible explanations for the comparative value found in our study may include gender variations and/or the influence of FMS comorbidities.

As indicated above, the inclusion of BMI in our model substantially attenuated the association of FMS to CRP, suggesting BMI may have largely explained the elevated CRP levels in FMS; this was second to the inclusion of comorbidities. Obesity is a major determinant of elevated CRP in multiple populations [50, 51]. Two studies to date have considered the potential contribution of BMI to the profile of CRP in FMS, with one demonstrating a strong and positive correlation [24] while another demonstrated overweight women with FMS had a higher mean CRP compared to normal weight women with FMS (*N* = 25; M = 3.1 ± 1.4 mg/L and M = 1.0 ± 0.8 mg/L, respectively). Additionally, in agreement with our study, others have suggested a mediating effect of BMI on the relation between CRP and FMS; FMS symptom improvement has followed weight loss among several FMS cases [52], and a longitudinal study revealed regular exercise and maintenance of body weight lowered the risk of FMS [53]. Additionally, one case-control study found that after adjustment for demographic factors, BMI was the only significant contributor in a model exploring the relation between CRP and FMS (*r* = .062, *p* < 0.001) [24].

Ours is the first study to assess the contribution of multiple comorbidities on the relation of CRP and FMS. Comorbidities characterized by pain may contribute to the development of FMS; shared pain mechanisms between FMS and other conditions with a similar underlying pathophysiology (e.g., tension headache) or as a comorbidity characterized by inflammation or ongoing peripheral damage (e.g., autoimmune disorders and osteoarthritis) and FMS have recently been explored [4, 39, 54]. Our study found that as the number of comorbidities increased, the odds of FMS increased as well.

Additionally, the presence of sleep impairment and mood disturbance modestly attenuated, but did not eliminate, the association between CRP and FMS. No existing studies have examined the role of sleep impairment on this relationship. We found no evidence supporting mood disorder as an effect modifier, similar to a study finding where CRP level did not differ by psychiatric status among those with FMS [26]. The influence of sleep impairment and mood disturbance on the relation between CRP and FMS in our study likely reflects a bidirectional relationship.

The prevalence rate of FMS in our study (2.1%) mirrored recent estimates for the U.S. population [3, 55]. Consistent with previous studies, we found the likelihood of FMS diagnosis was increased in middle-aged females [3, 5]. However, in contrast to findings from two large studies [3, 56], those who were married or reported higher levels of education were more likely to report FMS. Females who had experienced menopause were 1.8 times more likely to have FMS than those who had not; however, a lack of epidemiological research exists in the topical area of sex hormones, neurotransmitters, and FMS. We observed no significant association between age at menarche or parity and FMS, unlike a German case-control study of 653 middle-aged women (FMS cases = 36) which found, after controlling for age, those with FMS had significantly later menarche and were less likely to have ever been pregnant [57].

In agreement with previous cross-sectional and longitudinal studies [3, 53, 58], BMI was strongly and positively related to reported FMS in this large U.S. population. Additionally, higher BMI has been associated with an increased risk of FMS after adjustment for mood and/or other health factors [3, 53], including familial FMS diagnosis [58]. Likewise, consistent with findings from a recent cross-sectional study of a nationally representative sample of U.S. adults (*N* = 8446, FMS cases = 201) [3] FMS showed significant positive associations to multiple comorbid conditions in our study after adjustment for demographic and lifestyle factors; these included cardiovascular disease, rheumatoid arthritis and other autoimmune disorders, kidney, respiratory and liver diseases, and frequent headache. Osteoarthritis was also associated with a nearly 4-fold likelihood of FMS, dissimilar from a large, national analysis of U.S. electronic medical records (*N* = 587,961, FMS cases = 4296) of only a (unadjusted) 2-fold likelihood [59]. In contrast to other large studies of U.S. adults [3, 59], diabetes was not associated with FMS in this sample of adults.

The strengths of this study were its high response rate, population-based design, and large sample size; this was the largest comprehensive community study conducted to date in the Appalachian region of the U.S. We were able to control for a large number of potential confounders, including comorbid conditions. Misclassification of CRP was unlikely due to standardized assay procedures used.

This study targeted a population of predominantly white Appalachian adults in the U.S., potentially limiting generalizability. Possible misclassification of FMS may have occurred depending on time the participant was diagnosed and the physician’s awareness and use of the American College of Rheumatology classification criteria for FMS diagnosis, first established in 1990. In particular, poor or incomplete recognition of FMS by healthcare providers may have led to under-ascertainment [60]. However, such under-ascertainment would be expected to bias the observed associations toward the null, and thus would indicate that the magnitudes of relationships in this study are possibly stronger than those which we reported. FMS, in addition to most other assessed health conditions in this cross-sectional survey, was based on self-report without medical chart review, possibly leading to response or misclassification bias. To our knowledge, no clinical validation study has assessed the agreement between self-report and medical record-verified data to understand how patients accept and report their FMS diagnosis, especially in the presence of targeted interventions. Additionally, our assessment of the potential relationship between CRP and FMS could be strengthened by inclusion of symptom onset and response to interventions, for which data was not available.

Unmeasured confounding may have also contributed to our findings, although our ability to control for a large number of both known and potential risk factors for FMS diminishes this possibility. Our study also lacked specific information on certain conditions previously linked to FMS, including sleep apnea and irritable bowel syndrome. Although contact with former residents of counties used for study inclusion was attempted, some may not have participated in the study, possibly introducing sampling bias. Lastly, we cannot draw any conclusions regarding causality due to the lack of a temporal component in our cross-sectional study design.

In this large cross-sectional study, we observed a significant, positive association between serum CRP and diagnosed FMS, which was largely explained by elevated BMI and chronic comorbid conditions. Adjustment for sleep and mood disturbance only modestly attenuated this association, suggesting that BMI and chronic comorbid conditions may largely account for elevated CRP levels among FMS patients over the presence of sleep and mood disturbance. While the clinical utility of CRP for FMS diagnosis remains elusive at best, the current work contributes to existing literature by more appropriately assigning responsibility of inflammatory processes often present in those with FMS to co-occurring chronic conditions and other factors associated with chronic disease. Further prospective research is needed to determine the relation of CRP and other inflammatory markers to the development and progression of FMS in the presence of the potentially complex roles of obesity and comorbidities.

## Conclusion

Findings from this large cross-sectional study indicate the significant positive cross-sectional association of Fibromyalgia to serum CRP may be explained, in part, by factors such as BMI, comorbidity, impaired mood, and sleep disturbance. Prospective research is needed to confirm this, and better clarify the potential mediating influences on the relationship between Fibromyalgia and CRP.

## Abbreviations

BMI: Body Mass Index
CRP: C-reactive protein
FMS: Fibromyalgia syndrome
PFOA: Perfluorooctanoic Acid
WVU: West Virginia University
